# First-line atezolizumab/bevacizumab or durvalumab/tremelimumab in advanced hepatocellular carcinoma: a real world, multicenter retrospective study

**DOI:** 10.1093/oncolo/oyaf286

**Published:** 2025-09-18

**Authors:** Ioannis Kournoutas, Paulina Marell, Jennifer Gile, Anina Peersen, Priyanshi Shah, Kyle VanDommelen, Suneel D Kamath, Garima Gupta, Mehmet Akce, Ju Dong Yang, Pin-Jung Chen, Nikolas Naleid, Amit Mahipal, Nicole Peterson, Vaibhav Sahai, Wen Wee Ma, Zhaohui Jin, Thorvardur Halfdanarson, Lionel Fonkoua Kankeu, Leslie A Washburn, Caitlin B Conboy, Michael Torbenson, Ajit Goenka, Scott Thompson, Sudhakar K Venkatesh, Patrick Starlinger, Lewis Roberts, Gregory J Gores, Hani Babiker, Daniel Ahn, Mitesh Borad, Tanios Bekaii-Saab, Aminah Jatoi, Robert R McWilliams, Fang-Shu Ou, Nguyen H Tran

**Affiliations:** Department of Medicine, Mayo Clinic, Rochester, MN 55905, United States; Department of Medicine, Mayo Clinic, Rochester, MN 55905, United States; Department of Oncology, Mayo Clinic, Rochester, MN 55905, United States; Division of Clinical Trials and Biostatistics, Mayo Clinic, Rochester, MN 55905, United States; Department of Oncology, Mayo Clinic, Rochester, MN 55905, United States; Department of Hematology/Oncology, Cleveland Clinic Cancer Center, Cleveland, OH 44195, United States; Department of Hematology/Oncology, Cleveland Clinic Cancer Center, Cleveland, OH 44195, United States; Division of Hematology/Oncology, University of Alabama, Birmingham, AL 35294, United States; Division of Hematology/Oncology, University of Alabama, Birmingham, AL 35294, United States; Karsh Division of Gastroenterology and Hepatology, Cedars-Sinai Medical Center, Los Angeles, CA 90048, United States; Karsh Division of Gastroenterology and Hepatology, Cedars-Sinai Medical Center, Los Angeles, CA 90048, United States; Department of Oncology, University Hospitals Seidman Cancer Center and Case Western Reserve University, Cleveland, OH 44106, United States; Department of Oncology, University Hospitals Seidman Cancer Center and Case Western Reserve University, Cleveland, OH 44106, United States; Division of Medical Oncology, University of Michigan, Ann Arbor, MI 48019, United States; Division of Medical Oncology, University of Michigan, Ann Arbor, MI 48019, United States; Department of Hematology/Oncology, Cleveland Clinic Cancer Center, Cleveland, OH 44195, United States; Department of Oncology, Mayo Clinic, Rochester, MN 55905, United States; Department of Oncology, Mayo Clinic, Rochester, MN 55905, United States; Department of Oncology, Mayo Clinic, Rochester, MN 55905, United States; Department of Oncology, Mayo Clinic, Rochester, MN 55905, United States; Department of Oncology, Mayo Clinic, Rochester, MN 55905, United States; Department of Laboratory Medicine and Pathology, Mayo Clinic, Rochester, MN 55905, United States; Department of Radiology, Mayo Clinic, Rochester, MN 55905, United States; Department of Radiology, Mayo Clinic, Rochester, MN 55905, United States; Department of Radiology, Mayo Clinic, Rochester, MN 55905, United States; Department of Surgery, Mayo Clinic, Rochester, MN 55905, United States; Division of Gastroenterology, Mayo Clinic, Rochester, MN 55905, United States; Division of Gastroenterology, Mayo Clinic, Rochester, MN 55905, United States; Division of Hematology/Oncology, Mayo Clinic, Jacksonville, FL 32224, United States; Division of Hematology/Oncology, Mayo Clinic, Phoenix, AZ 85259, United States; Division of Hematology/Oncology, Mayo Clinic, Phoenix, AZ 85259, United States; Division of Hematology/Oncology, Mayo Clinic, Phoenix, AZ 85259, United States; Department of Oncology, Mayo Clinic, Rochester, MN 55905, United States; Department of Oncology, Mayo Clinic, Rochester, MN 55905, United States; Division of Clinical Trials and Biostatistics, Mayo Clinic, Rochester, MN 55905, United States; Department of Oncology, Mayo Clinic, Rochester, MN 55905, United States

**Keywords:** advanced hepatocellular carcinoma, atezolizumab, bevacizumab, durvalumab, tremelimumab, immunotherapy

## Abstract

**Background:**

Unresectable hepatocellular carcinoma (uHCC) is a leading cause of cancer death. FDA-approved first-line systemic therapies include atezolizumab/bevacizumab (atezo/bev) and durvalumab/tremelimumab (durva/treme); however, there is a lack of comparative data.

**Methods:**

We reviewed outcomes of patients with uHCC who initiated atezo/bev or durva/treme between 2017 and 2024, across six institutions. Overall survival (OS) and time to treatment discontinuation (TTD) were analyzed using the Kaplan–Meier and Cox models, adjusting for baseline characteristics.

**Results:**

Four hundred fifty-two uHCC pts were included. Median age: 68 years; 77% male; 81% white. Most common etiologies were viral hepatitis (38.9%) and metabolic dysfunction-associated steatohepatitis (19.5%). Disease progression was the primary reason for treatment discontinuation, atezo/bev (56%) and durva/treme (42%). Outcomes were not statistically significant (median OS [month, m]: 14.0 vs 14.6 [*P* = .66]; median TTD [m]: 4.9 vs 3.9 [*P* = .42] for atezo/bev vs durva/treme). Outcomes were significantly different between Child-Pugh classes (CP: A, B7, B8/9, C) respectively, median OS(m): 19.0, 6.1, 5.1, 2.0 (*P* < .001); median TTD(m): 6.1, 2.3, 3.0, 1.3 (*P* < .001).

**Conclusions:**

In this real-world study of uHCC, no significant difference in clinical outcomes was observed between atezo/bev and durva/treme in the first-line setting. CP scores were a key prognostic variable with both regimens.

Implications for practice This study offers real-world comparative data on two first-line regimens in uHCC. As multiple first-line regimen combinations emerge, assessment of differences in efficacy, safety, and patient selection outside of clinical trials remain an unmet need. These findings may help guide treatment decisions, particularly in settings where toxicity, comorbidities, or resource constraints influence regimen choice.

## Introduction

Hepatocellular carcinoma (HCC) accounts for over 80% of primary liver cancer and is the third leading cause of cancer-related death worldwide.^1^ Immune-checkpoint inhibitors (ICIs) have revolutionized the treatment of numerous solid-organ cancers including HCC. By targeting cell-surface receptors such as cytotoxic T-lymphocyte-associated protein 4 (CTLA-4) and programmed cell death protein 1 (PD-1) or its ligand PD-L1—receptors upregulated by tumor cells to evade immune surveillance—these agents allow the immune system to recognize and destroy cancer cells.^2^ Since 2020, two pivotal trials have led to the U.S. Food and Drug Administration (FDA) approval of ICIs in the first-line setting.

The IMbrave150 trial (2020) was a phase III clinical trial that evaluated atezolizumab (PD-L1 inhibitor) in combination with bevacizumab (vascular endothelial growth factor- [VEGF] inhibitor) compared to sorafenib in unresectable HCC.^3^ Overall survival (OS) was improved with atezolizumab + bevacizumab (atezo/bev) at 12 months (m) (67.2% vs 54.6%), and an improvement in median PFS by 2.5 m (6.8 vs 4.3 m).^3^ Extended follow up of the trial participants demonstrated a median overall survival of 19.2 m compared to 13.4 m in the sorafenib group.^4^ The IMbrave150 trial led to the establishment of atezo/bev as the new standard of care for patients with unresectable HCC (uHCC).

The phase III HIMALAYA trial (2022) sought to investigate the efficacy of a single priming-dose of tremelimumab (CTLA-4 inhibitor) followed by durvalumab (PD-L1 inhibitor), compared to sorafenib monotherapy.^5^ Durvalumab + tremelimumab (durva/treme) yielded a median OS of 16.4 m, compared to 13.8 m with sorafenib.^5^ The survival benefits observed in HIMALAYA and with the IMbrave150 trial have led to changes in treatment guidelines from many societies including the National Comprehensive Cancer Network (NCCN), American Society of Clinical Oncology (ASCO), European Society for Medical Oncology (ESMO), and the Pan-Asian Guidelines Adaptation (PAGA).^6–9^

Currently, there exists a paucity of data comparing atezo/bev and durva/treme performances in the real-world setting. A network meta-analysis of nine randomized control trials (RCT’s) of first-line systemic agents in uHCC suggested that atezo/bev may offer a net health benefit in terms of OS compared to other first-line agents in uHCC including durva/treme.^10^ This analysis, however, was limited by heterogeneity in patient characteristics and excluded patients with advanced liver disease (Child Pugh class B).

In the present manuscript, we evaluated and characterized the outcomes of patients treated with first-line atezo/bev and durva/treme in uHCC, leveraging real-world data involving six tertiary cancer centers across the United States. We also sought to evaluate the prognostic significance of Child-Pugh class and body mass index (BMI) at the time of treatment initiation.

## Methods

### Overview

We retrospectively reviewed the electronic health records of patients who initiated first-line atezo/bev or durva/treme for uHCC between March 1, 2017 and February 29, 2024 across 6 institutions: Mayo Clinic and the Mayo Clinic Health System (Arizona, Florida, Minnesota, Iowa, Wisconsin), Cleveland Clinic, University of Alabama, Cedars-Sinai Medical Center (Los Angeles), University Hospitals Seidman Cancer Center (Cleveland), and University of Michigan. This study was reviewed and approved by the centralized Mayo Clinic institutional review board (IRB 22-012772). The data were locked for analysis on May 16, 2024.

### Eligibility

Patient inclusion criteria for data extraction consisted of the following: (1) ≥18 yrs at time of cancer diagnosis, (2) radiologically and/or pathologically confirmed evidence of HCC, (3) initiation of systemic treatment within the aforementioned date range, and (4) received care at the institution (including at least one cycle of systemic treatment or a minimum of two clinic follow up visits). Patients were excluded if they did not receive atezo/bev or durva/treme first line, received either regimen in conjunction with another experimental trial drug or intervention, or received either regimen while simultaneously receiving treatment for a malignancy other than HCC.

### Data acquisition

At Mayo Clinic, Advanced Text Explorer, an automated natural language processing tool, was used to search the electronic medical records. Key search terms consisted of: “hepatocellular carcinoma” OR “liver cancer,” AND “atezolizumab” OR “bevacizumab” OR “durvalumab” OR “tremelimumab.” Three investigators (IK, PM, and JG) reviewed the medical records and abstracted key data elements. Other participating institutions utilized similar patient search tools to pull and survey respective medical records.

The variable of interest for this study was atezo/bev vs durva/treme where durvalumab monotherapy is also included in the durva/treme group.

Variables collected at time of treatment initiation included: age, sex, race, height/weight, etiology of HCC, Child-Pugh (CP) score, Barcelona-Clinic Liver Cancer (BCLC) stage, and albumin-bilirubin (ALBI) grade, pre-treatments received (including transarterial chemoembolization, microwave ablation, stereotactic radiotherapy, radioembolization), cirrhosis, extrahepatic spread or macrovascular invasion, date of treatment initiation/discontinuation, reason for discontinuation, and best treatment response. Macrovascular invasion was defined as involvement of the main portal vein or inferior vena cava (IVC). Response criteria were categorized as complete response (CR), partial response (PR), stable disease (SD), or progressive disease (PD) as defined by response evaluation criteria in solid tumors (RECIST v1.1).^11^

### Study endpoints

Primary endpoints included overall survival (OS), and time to treatment discontinuation (TTD). Overall survival was defined as the time from the initiation of first-line systemic treatment to death from any cause. For patients who were alive or lost to follow-up, the patient was censored at the date of last follow-up. Time to treatment discontinuation was defined as the time from initiation of first-line systemic treatment to cessation of treatment due to any cause (including death). If the patient was still on treatment at the time of data extraction, the patient was censored at the date of last follow-up.

The objective response rate (ORR) was defined as the proportion of patients with partial response (PR) or complete response (CR). The disease control rate (DCR) was defined as the proportion of patients with CR, PR, or stable disease (SD).

### Statistical analysis

Baseline clinical characteristics were compared between the two treatment groups. Continuous variables were presented with median, interquartile range, and range; categorical variables were presented with count and percentage. Univariate comparisons were performed using Wilcoxon rank-sum test for continuous variables and Pearson Chi-square tests for categorical variables. OS and TTD distributions were estimated by Kaplan–Meier curves and the difference between groups was assessed by log-rank test. Multivariable analyses were performed for time-to-event outcomes using Cox proportional hazard models; results were reported as hazard ratios (HR) with 95% confidence intervals (CI). Subgroup analyses were conducted using Cox models that included treatment groups, the subgroup of interest, and their interaction. The subgroup analyses were multivariable adjusted, and the *P*-value of the interaction term was calculated using the likelihood ratio test. Multivariable analyses for binary outcomes were conducted using logistic regression; results were reported as odds ratios (OR) with 95% CI. Variables included in the multivariable models are age (continuous), sex (female vs male), race (non-white vs white), etiology (viral vs non-viral), Child-Pugh class (A vs B7 vs B8/9 vs C), albumin/bilirubin interpretation (A1 vs A2 vs A3), cirrhosis (yes vs no), ECOG performance status (0 vs 1 vs 2/3), and prior selective internal radiation therapy (SIRT, yes vs no). For logistic models, we did not adjust for albumin/bilirubin interpretation and collapsed Child-Pugh class as A vs B/C due to the small number of patients who experienced objective response in the durva/treme group. For categorical variables with more than 2-levels, the overall strength of association was quantified by a chi-square test using linear contrast. Sensitivity analysis was conducted by excluding patients who received durvalumab monotherapy. A *P*-value of <.05 is considered statistically significant, except for the test of interaction where a *P*-value < .1 is considered statistically significant. All statistical analyses were performed using SAS (version 9.4; SAS Institute, Cary, NC, USA).

## Results

### Patients and baseline characteristics

**Figure 1.**
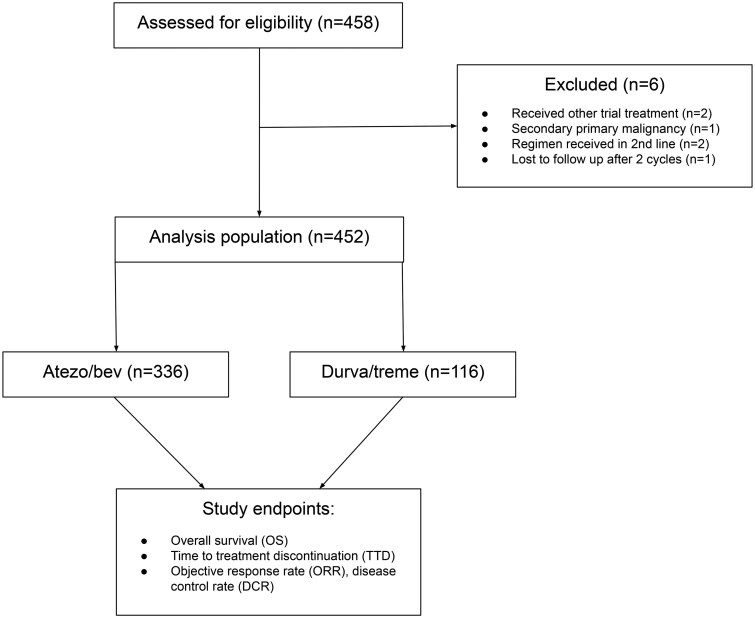
Consort diagram. Atezo/bev: atezolizumab/bevacizumab; durva/treme: durvalumab/tremelimumab.

A total of 452 patients met eligibility criteria: atezo/bev (*n* = 336), durva/treme (*n* = 116) (Figure 1). Median (interquartile range) follow-up time was 17.1 (8.7-25.0) months. Baseline patient and disease characteristics are summarized in Table 1.

**Table 1.** Baseline characteristics at treatment initiation.

	Atezo/Bev	Durva/Treme	Total	*P*-value
	(*N* = 336)	(*N* = 116)	(*N* = 452)	
**Age**				.053^a^
***N***	336	116	452	
** Mean (SD)**	66.5 (10.18)	68.4 (9.36)	67.0 (10.00)	
** Median**	67.0	69.5	68.0	
** IQR**	61.0, 73.0	63.0, 74.0	62.0, 74.0	
** Range**	23.0, 90.0	26.0, 90.0	23.0, 90.0	
**Sex, *n* (%)**				.937^b^
** Male**	259 (77.1%)	89 (76.7%)	348 (77.0%)	
** Female**	77 (22.9%)	27 (23.3%)	104 (23.0%)	
**Race, *n* (%)**				.0003^b^
** White**	287 (85.4%)	81 (69.8%)	368 (81.4%)	
** Asian**	13 (3.9%)	10 (8.6%)	23 (5.1%)	
** African American**	20 (6.0%)	17 (14.7%)	37 (8.2%)	
** Unknown or Not Reported**	10 (3.0%)	1 (0.9%)	11 (2.4%)	
** Hispanic/Latino**	6 (1.8%)	7 (6.0%)	13 (2.9%)	
**BMI (kg/m^2^)**				.716^a^
***N***	332	116	448	
** Mean (SD)**	28.9 (6.38)	28.9 (6.05)	28.9 (6.29)	
** Median**	28.1	28.3	28.2	
** IQR**	24.3, 32.5	25.0, 32.6	24.5, 32.5	
** Range**	16.6, 61.7	18.1, 49.8	16.6, 61.7	
**Obesity status, *n* (%)**				.696^b^
** BMI < 30 kg/m^2^**	205 (61.7%)	74 (63.8%)	279 (62.3%)	
** BMI >= 30 kg/m^2^**	127 (38.3%)	42 (36.2%)	169 (37.7%)	
** Missing**	4	0	4	
**Etiology, *n* (%)**				.112^b^
** Any Viral**	125 (37.2%)	51 (44.0%)	176 (38.9%)	
** MASH Alone**	60 (17.9%)	28 (24.1%)	88 (19.5%)	
** Alcohol Alone**	32 (9.5%)	10 (8.6%)	42 (9.3%)	
** Combination/Other**	37 (11.0%)	11 (9.5%)	48 (10.6%)	
** Unknown**	82 (24.4%)	16 (13.8%)	98 (21.7%)	
**Prior surgery, *n* (%)**				.359^b^
** No**	300 (89.3%)	107 (92.2%)	407 (90.0%)	
** Yes**	36 (10.7%)	9 (7.8%)	45 (10.0%)	
**Prior ablation, *n* (%)**				.239^b^
** No**	290 (86.3%)	105 (90.5%)	395 (87.4%)	
** Yes**	46 (13.7%)	11 (9.5%)	57 (12.6%)	
**Prior embolization, *n* (%)**				.531^b^
** No**	224 (66.7%)	81 (69.8%)	305 (67.5%)	
** Yes**	112 (33.3%)	35 (30.2%)	147 (32.5%)	
**Prior SIRT (Y90), *n* (%)**				.004^b^
** No**	306 (91.1%)	94 (81.0%)	400 (88.5%)	
** Yes**	30 (8.9%)	22 (19.0%)	52 (11.5%)	
**Cirrhosis, *n* (%)**				.0002^b^
** No**	95 (28.3%)	13 (11.2%)	108 (23.9%)	
** Yes**	241 (71.7%)	103 (88.8%)	344 (76.1%)	
**ECOG performance score *n* (%)**				.014^b^
** 0**	138 (41.7%)	30 (26.5%)	168 (37.8%)	
** 1**	165 (49.8%)	65 (57.5%)	230 (51.8%)	
** 2**	26 (7.9%)	17 (15.0%)	43 (9.7%)	
** 3**	2 (0.6%)	1 (0.9%)	3 (0.7%)	
** Missing**	5	3	8	
**BCLC stage, *n* (%)**				.012^b^
** A**	15 (4.5%)	3 (2.6%)	18 (4.0%)	
** B**	140 (41.8%)	31 (26.7%)	171 (37.9%)	
** C**	175 (52.2%)	81 (69.8%)	256 (56.8%)	
** D**	5 (1.5%)	1 (0.9%)	6 (1.3%)	
** Missing**	1	0	1	
**Extrahepatic spread present *n* (%)**				.399^b^
** No**	214 (63.9%)	69 (59.5%)	283 (62.7%)	
** Yes**	121 (36.1%)	47 (40.5%)	168 (37.3%)	
** Missing**	1	0	1	
**Macrovascular invasion present *n* (%)**				.498^b^
** No**	193 (57.6%)	71 (61.2%)	264 (58.5%)	
** Yes**	142 (42.4%)	45 (38.8%)	187 (41.5%)	
** Missing**	1	0	1	
**Child-Pugh at first line, *n* (%)**				.733^b^
** A**	234 (72.2%)	77 (67.5%)	311 (71.0%)	
** B7**	49 (15.1%)	17 (14.9%)	66 (15.1%)	
** B8**	19 (5.9%)	9 (7.9%)	28 (6.4%)	
** B9**	14 (4.3%)	6 (5.3%)	20 (4.6%)	
** C**	8 (2.5%)	5 (4.4%)	13 (3.0%)	
** Missing**	12	2	14	
**AFP (ng/mL)**				.228^a^
***N***	327	116	443	
** Mean (SD)**	9504.1 (36 492.55)	5940.9 (18547.35)	8571.1 (32 774.56)	
** Median**	73.8	118.8	90.0	
** IQR**	7.4, 1257.0	9.2, 1854.3	7.4, 1364.0	
** Range**	1.3, 369 500.0	2.0, 121 000.0	1.3, 369 500.0	
**ALBI grade at first line, *n* (%)**				.083^b^
** A1**	134 (40.6%)	34 (29.3%)	168 (37.7%)	
** A2**	172 (52.1%)	70 (60.3%)	242 (54.3%)	
** A3**	24 (7.3%)	12 (10.3%)	36 (8.1%)	
** Missing**	6	0	6	

a Wilcoxon Rank-Sum *P*-value.

b Chi-Square *P*-value.

Abbreviations: SD: standard deviation; IQR: interquartile range; Atezo/Bev: atezolizumab/bevacizumab; Durva/Treme: durvalumab/tremelimumab; BMI: body mass index; MASH: metabolic dysfunction-associated steatohepatitis; SIRT: selective internal radiation therapy; ECOG: Eastern cooperative oncology group; BCLC: Barcelona clinic liver cancer; AFP: alpha fetoprotein; ALBI: albumin-bilirubin.

Median age at the start of therapy was 68 [range: 23-90]; 77.0% of the total cohort was male. The racial composition for the atezo/bev group was white, non-Hispanic (85.4%), African American (6.0%), Asian (3.9%), Hispanic/Latino (1.8%), or unknown/unreported (3.0%); the racial composition of durva/treme was white, non-Hispanic (69.8%), African American (14.7%), Asian (8.6%), Hispanic/Latino (6.0%), or unknown/unreported (0.9%) (*P*-value < .001). Etiologies of HCC in the total cohort were viral hepatitis (38.9%), metabolic dysfunction associated steatohepatitis (MASH) (19.5%), alcohol (9.3%), multifactorial (10.6%), or unknown (21.7%). At the time of treatment initiation, the rate of cirrhosis was 71.7% in the atezo/bev cohort and 88.8% in the durva/treme cohort (*P*-value < .001). The frequency of extrahepatic spread at time of treatment initiation in the atezo/bev and durva/treme cohorts, respectively, was 36.1% and 40.5% (*P*-value = .399), and rate of macrovascular invasion at treatment initiation was 42.4% and 38.8%, respectively (*P*-value = .498). In the atezo/bev cohort, 4.5% had BCLC stage A, 41.8% B, 52.2% C, and 1.5% D; in the durva/treme cohort 2.6% had stage A, 26.7% B, 69.8% C, and 0.9% D (*P*-value = .012). Child-Pugh score(s) at treatment initiation in the atezo/bev cohort were (%): A (72.2%), B7 (15.1%), B8 (5.9%), B9 (4.3%), and C (2.5%); and in the durva/treme cohort were: A (67.5%), B7 (14.9%), B8 (7.9%), B9 (5.3%), and C (4.4%) (*P*-value = .733). Median body mass index (BMI) at treatment initiation was 28.2 kg/m^2^. A total of 169 participants (37.7%) had a BMI ≥30.0 kg/m^2^, 127 patients (38.3%) in the atezo/bev group, and 42 patients (36.2%) in the durva/treme group.

### Overall survival

**Figure 2.**
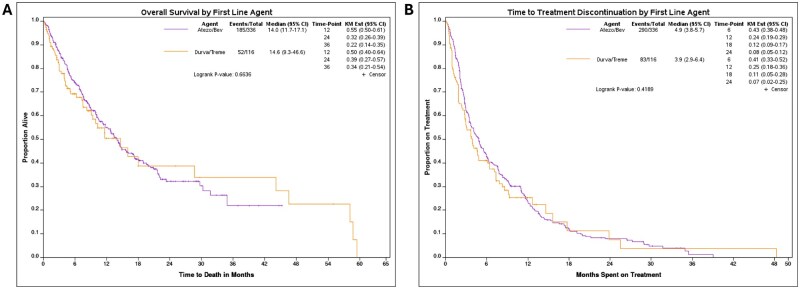
(A) Overall survival and (B) time to treatment discontinuation, by first-line agent. Atezo/bev: atezolizumab/bevacizumab; durva/treme: durvalumab/tremelimumab.

OS did not differ significantly between the two cohorts (Figure 2A). Median OS, in months, for atezo/bev and durva/treme were 14.0 (95% CI: 11.7-17.1) and 14.6 (95% CI: 9.3-46.6), respectively (*P*-value = 0.664).

### Time to treatment discontinuation

TTD also did not differ significantly between the cohorts (Figure 2B). Median TTD, in months, was 4.9 (95% CI: 3.8-5.7) in the atezo/bev cohort and 3.9 (95% CI: 2.9-6.4) in the durva/treme cohort (*P*-value = .419). Reasons for treatment discontinuation were similar between the two groups. The most common reasons were disease progression (53.1%), death or transition to hospice (18.2%), toxicity (11.0%), and functional decline (9.9%) (Table 2).

**Table 2.** Treatment response and discontinuation.

	Atezo/Bev	Durva/Treme	Total	
	(*N* = 336)	(*N* = 116)	(*N* = 452)	
**Best response, *n* (%)**				0.379^a^
** CR**	8 (2.4%)	5 (4.6%)	13 (2.9%)	
** PR**	80 (23.9%)	19 (17.4%)	99 (22.3%)	
** SD**	118 (35.2%)	37 (33.9%)	155 (34.9%)	
** PD**	86 (25.7%)	29 (26.6%)	115 (25.9%)	
** Not evaluable^b^**	43 (12.8%)	19 (17.4%)	62 (14.0%)	
** Missing^c^**	1	7	8	
**Objective response, *n* (%)**				0.375^a^
** Yes**	88 (26.3%)	24 (22.0%)	112 (25.2%)	
** No**	247 (73.7%)	85 (78.0%)	332 (74.8%)	
** Missing^c^**	1	7	8	
**Disease control response, *n* (%)**				0.306^a^
** Yes**	206 (61.5%)	61 (56.0%)	267 (60.1%)	
** No**	129 (38.5%)	48 (44.0%)	177 (39.9%)	
** Missing^c^**	1	7	8	
**Off treatment reason, *n* (%)**				0.030^a^
** Disease Progression**	163 (56.2%)	35 (42.2%)	198 (53.1%)	
** Toxicity**	30 (10.3%)	11 (13.3%)	41 (11.0%)	
** Death/Hospice**	55 (19.0%)	13 (15.7%)	68 (18.2%)	
** Complete Response**	2 (0.7%)	1 (1.2%)	3 (0.8%)	
** Liver Transplant**	1 (0.3%)	1 (1.2%)	2 (0.5%)	
** Patient Withdrawal**	7 (2.4%)	6 (7.2%)	13 (3.5%)	
** Secondary Resection**	1 (0.3%)	0 (0.0%)	1 (0.3%)	
** Other/Unknown**	9 (3.1%)	1 (1.2%)	10 (2.7%)	
** Functional Decline**	22 (7.6%)	15 (18.1%)	37 (9.9%)	
** Missing**	46	33	79	

a Chi-square *P*-value.

b 43 Atezo/Bev and 19 Durva/Treme patients did not have a post-baseline restaging scan before off-treatment.

c 1 Atezo/Bev and 7 Durva/Treme patients were still on-treatment without any re-staging scan at the time of data extraction.

Abbreviations: CR: complete response; PR: partial response; SD: stable disease; PD: progressive disease; Atezo/Bev: atezolizumab/bevacizumab; Durva/Treme: durvalumab/tremelimumab.

### Response rates

Similarly, ORR and DCR were not significantly different between the cohorts (Table 2). Atezo/bev patients (26.3%) and 22.0% of durva/treme achieved ORR (*P*-value = .375). Atezo/bev (61.5%) patients and 56.0% of durva/treme patients achieved DCR (*P*-value = .306).

### Multivariable analysis

Multivariable analysis of OS and TTD found no difference between atezo/bev and durva/treme for either OS (HR 0.93; 95% CI: 0.66-1.32; *P* = .68) or TTD (HR 1.07; 95% CI: 0.81-1.40; *P* = .64); these results are summarized, respectively, in Tables S1 and S2. Sex was significantly associated with OS and TTD, showing that female patients had shorter overall survival (HR 1.38, 95% CI: 1.01-1.89; *P* = .04) and stayed on treatment for a shorter time (HR 1.30, 95% CI: 1.01-1.68; *P* = .04) compared to males. Higher Child-Pugh class was correlated with worse OS and TTD (overall *P* < .001 and =0.003, respectively). For OS, class B7, B8/9, and C had a HR of 2.00 (95% CI: 1.37-2.94; *P* < .001), 2.18 (95% CI: 1.37-3.47; *P* = .001), and 6.10 (95% CI: 2.86-12.98; *P* < .001), respectively, compared to class A. For TTD, class B7, B8/9, and C had a HR of 1.58 (95% CI: 1.51-2.18; *P* = .005), 1.61 (95% CI: 1.08-2.40; *P* = .02), and 2.79 (95% CI: 1.36-5.71; *P* = .005), respectively, compared to class A. Higher ALBI grade had a similar effect when compared to grade A1 (OS overall *P* = .0009 and TTD overall *P* = .01): for OS grade A2 had a HR of 1.99 (95% CI: 1.38-2.86; *P* < .001) and A3 2.24 (95% CI: 1.20-4.18; *P* = .01); and for TTD A2 had a HR of 1.51 (95% CI: 1.16-1.98; *P* = .002) and A3 1.51 (95% CI: 0.89-2.57; *P* = .13). Worse ECOG performance status was associated with shorter survival (overall *P* = .0003), HR for ECOG 1 and 2/3 are 1.41 (95% CI: 1.05-1.90; *P* = .02) and 2.59 (95% CI: 1.62-4.13; *P* < .001), respectively, compared to ECOG 0; but it was not associated with TTD (overall *P* = .14), HR for ECOG 1 and 2/3 are 0.95 (95% CI: 0.75-1.19; *P* = .64) and 1.39 (95% CI: 0.94-2.05; *P* = .10). Receiving prior SIRT, in contrast, was not associated with OS, HR 0.77 (95% CI: 0.49-1.20; *P* = .24) but was associated with longer TTD, HR 0.65 (95% CI: 0.46-0.93; *P* = .02).

Multivariable analysis of response rates found no significant difference between the treatments in either ORR (OR 0.72; 95% CI: 0.41-1.27; *P* = .26) or DCR (OR: 0.74; 95% CI: 0.46-1.20; *P* = .23); these results are summarized in Tables S3 and S4. However, patients with Child-Pugh class B and C are less likely to achieve ORR and DCR (OR: 0.38, 95% CI: 0.21-0.69, *P* = .002 for ORR; OR: 0.47, 95% CI: 0.30-0.76, *P* = .002 for DCR) compared to patients with Child-Pugh class A. Cirrhosis is significantly associated with higher DCR (OR 1.74; 95% CI: 1.04-2.92; *P* = .04). Prior SIRT is also significantly associated with higher DCR (OR 2.39; 95% CI: 1.15-4.97; *P* = .02).

### Sensitivity analysis

The associations between treatments and clinical outcomes remain the same after excluding patients receiving durvalumab monotherapy (Tables S5-S8).

### Subgroup analysis

**Figure 3.**
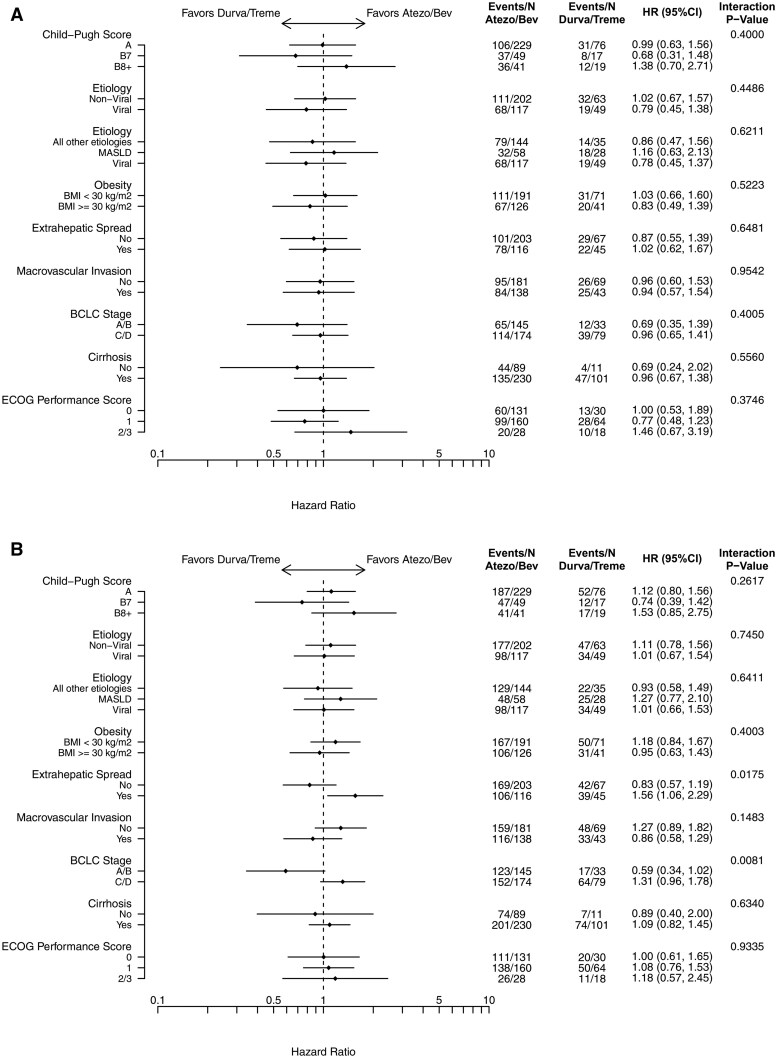
Subgroup analysis of (A) overall survival and (B) time to treatment discontinuation.

There was no significant difference in OS between the treatments in all subgroups examined (Figure 3A). Similarly, there was no significant difference in TTD between the treatments in most subgroups examined (Figure 3B), except for extrahepatic spread (interaction *P* = .018) and BCLC stage (interaction *P* = .008). Patients with no extrahepatic spread treated with durva/treme had a numerically longer duration on treatment compared to those treated with atezo/bev (HR 0.83, 95% CI: 0.57-1.19); in contrast, patients with extrahepatic spread treated with durva/treme had a shorter duration on treatment compared to those treated with atezo/bev (HR 1.56, 95% CI: 1.06-2.29). Additionally, patients with BCLC stage A/B treated with durva/treme had a numerically longer duration on treatment compared to those treated with atezo/bev (HR 0.59, 95% CI: 0.34-1.02); on the other hand, patients with BCLC stage B/C treated with durva/treme had a numerically shorter duration on treatment compared to those treated with atezo/bev (HR 1.31, 95% CI: 0.96-1.78).

### Child-Pugh class and obesity

**Figure 4.**
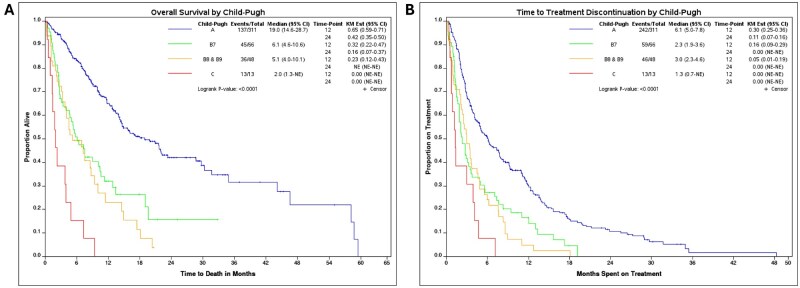
(A) Overall survival and (B) time to treatment discontinuation, by Child-Pugh class.

Given the demonstrated significance of Child-Pugh class and the absence of a statistically significant effect of regimen on the outcome variables in the multivariable analysis, a small exploration into the effect of Child-Pugh class on clinical outcomes was performed. Median OS in months for Child-Pugh class A, B7, B8/9, and C were 19.0 (95% CI: 14.6-28.7), 6.1 (95% CI: 4.6-10.6), 5.1 (95% CI: 4.0-10.1), and 2.0 [95% CI: 1.3-Not estimable (NE)], respectively (*P* < .001, Figure 4A). Median TTD in months for class A, B7, B8/9, and C were 6.1 (95% CI: 5.0-7.8), 2.3 (95% CI: 1.9-3.6), 3.0 (95% CI: 2.3-4.6), and 1.3 (95% CI: 0.7-NE), respectively (*P* < .001, Figure 4B). ORR for Child-Pugh class A, B7, B8/9, and C were 29.6%, 16.9% 16.7%, and 0%, respectively. DCR for Child-Pugh class A, B7, B8/9, and C were 65.1%, 47.7%, 50%, and 38.5%, respectively.

Additional analysis was performed to assess the impact of obesity on clinical outcomes. Median OS was not significantly different for those with obesity (≥30 kg/m^2^; 13.8 months, 95% CI: 10.3-19.6) compared to those who were non-obese (<30 kg/m^2^; 13.4 months, 95% CI: 11.3-16.0; *P* = .93; Figure S1A). Median TTD also was not significantly different for the elevated BMI cohort (4.1 months, 95% CI: 3.4-6.2) compared to non-obese BMI (4.7 months, 95% CI: 3.7-5.7; *P* = .66; Figure S1B). Similarly, no significant differences were found in the rates of objective response (26.7% vs 22.9%, *P* = .37) and disease control (60.1% vs 60.8%, *P* = .87) between the non-obese and obese cohorts. The lack of association between obesity status and clinical outcomes remained after multivariable adjustment (Tables S9-S12).

## Discussion

In this multi-institutional retrospective cohort study, we report the real-world experience of patients with unresectable HCC who received first-line systemic therapy with atezo/bev or durva/treme. This is the largest cohort to date evaluating both regimens in the United States. Our analysis found that the difference in median OS was not statistically significant between the atezo/bev and durva/treme cohorts (14.0 m and 14.6 m, respectively). These results are consistent with a previous pooled meta-analysis which found a median OS of 14.5 months for first-line atezo/bev across nine studies with Child-Pugh class A & B disease.^12^

In our study, the ORR and DCR in the atezo/bev cohort were 26.3% and 61.5%, respectively, which are comparable to IMbrave150 (27.3% and 73.6%, respectively) per RECIST v1.1 criteria.^3^ The frequency of patients discontinuing atezo/bev due to toxicity was slightly higher than IMbrave150 (10.3% vs 7.0%).^3^ Given the heterogeneous patient population with variable Child-Pugh classes included in this study, we would expect slightly higher discontinuation rate due to toxicity.

There remains a need for expanded real-world evidence for durva/treme in patients with unresectable HCC as it is a more recently approved regimen. Like other phase III trials, HIMALAYA exclusively enrolled patients with Child-Pugh class A liver disease and excluded patients with main portal vein thrombosis. Taking this into account, the results of this cohort are comparable with those in HIMALAYA in terms of OS (14.6 m vs 16.4 months), ORR (22.0% vs 20.1%), and DCR (56.0% vs 60.1%).^5^ Treatment-related adverse events leading to discontinuation of durva/treme were similar (13.3% vs 13.7%).^5^ A recent 2024 real-world retrospective study of durva/treme in both first- and later-line patient cohorts found a comparable ORR and DCR of 15.0% and 53.3%, respectively.^13^

Prior studies lack robust evidence regarding the use of immunotherapy and resultant clinical outcomes in patients with Child-Pugh class B and C disease. A 2020 study by Lee et al. demonstrated that, in a cohort of patients with HCC treated with nivolumab or pembrolizumab, Child-Pugh class was an independent risk factor that negatively correlated with OS.^14^ A large 2023 meta-analysis by Xie et al. looking at a cohort of HCC patients with Child-Pugh class A and B disease found that median OS in patients with Child-Pugh class B liver disease was 5.49 months—significantly lower than in patients with Child-Pugh class A status.^15^ In our study, median OS in Child-Pugh classes B and C were 6.1 months and 2.0 months, respectively. We further differentiated Child-Pugh B7, B8/9, and C which demonstrated a median OS of 6.1 m, 5.1 m, 2.0 m across regimens. In this particular cohort, outcomes in Child-Pugh class B7 were similar to the other Child-Pugh B classes; however, as there is was no control group and given the small sample size of Child-Pugh B7, B8/9, a definitive conclusion cannot be made as to the potential benefit of first-line therapy in this specific group. These findings in our study corroborate and are consistent with prior data on the prognostic significance of underlying liver dysfunction on OS in patients with HCC.^15–17^

The treatment landscape for patients with advanced HCC is rapidly evolving. Recently, the combination regimen ipilimumab (CTLA-4 inhibitor) and nivolumab (anti-PD-1 antibody) in the first-line setting (CheckMate-9DW) reported positive results showing median OS of 23.7 m vs 20.6 m compared to sorafenib/lenvatinib (HR 0.79, 95% CI (0.65-0.95); (*P* = .018)).^18^ The objective response rate for the combination was 36% compared to 13% for sorafenib/lenvatinib (*P* < .0001).^18^ The CARES-310 study evaluated the combination camrelizumab (anti-PD-1 antibody) and rivoceranib (anti-VEGF inhibitor) to sorafenib and demonstrated a median OS of 22.1 m vs 15.2 m (HR 0.62, 95% CI (0.49-0.80, *P* < .0001)).^19^ CheckMATE 9DW has already received FDA approval as another first-line option, and thus, treatment selection will depend on efficacy, toxicity profile, and individual patient characteristics. A real-world database (across institutions) curated to evaluate efficacy and toxicity among all first-line options will be critical in defining the optimal regimen upfront. Future studies to assess the optimal second line regimen following any of these first-line options will also be of high importance. Our institutional experience evaluating continuing ICIs versus tyrosine kinase inhibitors (TKIs) in the second line following up-front atezo/bev demonstrated no difference in OS and TTD.^20^

A large unmet need is the identification of potential biomarkers that best predict response to immunotherapy, and to better clarify which HCC patients are most likely to derive benefit with less toxicity. Biomarkers such as tumor mutational burden (TMB), PD-L1 expression, and tumor-infiltrating lymphocytes (TIL’s) have been previously investigated, however, none have been consistently validated or routinely adopted within clinical practice.^21^^,^^22^ MASH-HCC (previously NASH-HCC) was investigated across several mouse models, and MASH-affected livers were found to have an increased frequency of CD8 + PD1 + T cells compared to controls; treatment with immunotherapy paradoxically did not lead to tumor regression but instead exacerbated liver fibrosis and increased incidence of HCC in mice.^23^ A follow-up meta-analysis of patients with MASLD-HCC showed worsened overall survival compared to viral induced-HCC when treated with immune checkpoint blockade.^24^ Thus, MASH-HCC was speculated to be unresponsive to immunotherapy and may be a relevant clinical marker. However, several follow up studies have challenged these findings given the heterogeneity of the studies that were included in the meta-analysis; in addition, MASLD-HCC as an etiology is highly diverse and not purely reducible to MASH-HCC.^25^^,^^26^ Interestingly, the results of our present analysis suggest that viral versus non-viral etiologies did not have a significant effect on clinical outcomes. Lastly, a recent 2024 study by Shu et al. utilized a multiomic approach to evaluate the immunologic characteristics of intratumoral tertiary lymphoid structures that may better predict response to immunotherapy.^27^ This remains an area of great interest and exploration.

Another area of significant interest pertains to the role of obesity in both the development of HCC and response to systemic immunotherapy. Obesity plays a unique role in a subset of patients who go on to develop HCC. In one respect, obesity is directly implicated in the pathogenesis of HCC through the facilitation of hepatic inflammation and oxidative stress, which in turn may lead to the development of metabolic-dysfunction associated steatotic liver disease (MASLD) and MASH.^28^ MASLD affects nearly 25%-30% of the global population, and epidemiological estimates forecast a continued rise in incidence through 2030 corresponding to increasing rates of obesity and metabolic disease (eg, Type 2 diabetes mellitus).^29^^,^^30^ Conversely, there has been previously documented in the literature an “*obesity paradox*,” which refers to the association of obesity with improved clinical outcomes in several different types of cancers,^31–33^ nevertheless, the concept remains controversial.^34^ With reference specifically to unresectable HCC, a recent 2023 study by Uojima et al. found that lower adiposity correlated with shorter PFS and OS in patients receiving systemic immunotherapy; looking specifically at BMI, this study found significantly shorter PFS, but no difference in OS.^35^ In our study, patients with obese BMI (≥30 kg/m^2^) had similar clinical outcomes to patients with normal or overweight BMI (≤30 kg/m^2^) including median OS, median TTD, ORR, and DCR; none of the differences met the threshold for statistical significance. To better characterize which patients with HCC may benefit most from systemic therapy, future studies will need to continue to evaluate the prognostic significance of BMI in combination with other measures of body composition and indices of fat distribution such as central obesity and visceral versus subcutaneous fat.^36^

Our study has several limitations. First, as an observational (retrospective study), it is possible that there are additional confounders that have not been controlled for. We have made every attempt to include a comprehensive review of patient characteristics as appropriate and feasible in the real-world setting. Second, due to the heterogeneous and diverse clinical practice settings in which patients in this study received their care, granular details on toxicity, serious adverse events, grading of events, and incidence of crossover were not attainable. However, we were able to obtain the reasons for coming off treatment, and hence, were able to use this variable to inform the endpoint of TTD.

## Conclusion

In this large real-world multi-institutional retrospective cohort study of unresectable HCC, there were no statistically significant differences in overall survival, objective response, and time to treatment discontinuation between atezo/bev and durva/treme in the first-line setting. The absence of a statistically significant difference in time-to-event outcomes persisted even after completion of multivariable analysis. Clinical outcomes were significantly different between Child-Pugh classes (CP: A, B, C): respectively, OS: 19.0 m, 6.1 m, 2.0 m; TTD: 6.1 m, 2.7 m, 1.3 m. The present study may be considered by clinicians deciding between these two regimens in patients with HCC, although future investigations will be necessary to fully elucidate additional factors of interest, including real-world comparative side-effect profiles and quality of life indices.

## Supplementary Material

oyaf286_Supplementary_Data

## Data Availability

All data generated or analyzed during this study are included in this article and its online supplementary material files. Further inquiries can be directed to the corresponding author.

## References

[1] Sung H , Ferlay J, Siegel RL, et al Global cancer statistics 2020: GLOBOCAN estimates of incidence and mortality worldwide for 36 cancers in 185 countries. CA Cancer J Clin. 2021;71:209-249. 10.3322/caac.21660 33538338

[2] He X , Xu C. Immune checkpoint signaling and cancer immunotherapy. Cell Res. 2020;30:660-669. https://www.nature.com/articles/s41422-020-0343-4 32467592 10.1038/s41422-020-0343-4 PMC7395714

[3] Finn RS , Qin S, Ikeda M, et al Atezolizumab plus bevacizumab in unresectable hepatocellular carcinoma. N Engl J Med. 2020;382:1894-1905. 10.1056/nejmoa1915745 32402160

[4] Cheng A-L , Qin S, Ikeda M, et al Updated efficacy and safety data from IMbrave150: atezolizumab plus bevacizumab vs. sorafenib for unresectable hepatocellular carcinoma. J Hepatol. 2022;76:862-873. 10.1016/j.jhep.2021.11.030 34902530

[5] Abou-Alfa GK , Lau G, Kudo M, et al Tremelimumab plus durvalumab in unresectable hepatocellular carcinoma. NEJM Evid. 2022;1:EVIDoa2100070. 10.1056/evidoa2100070 38319892

[6] Referenced with Permission from the NCCN Clinical Practice Guidelines in Oncology (NCCN Guidelines^®^) for Guideline Name: Hepatocellular Carcinoma Version 2.*2024*. © National Comprehensive Cancer Network, Inc. 2024. Accessed August 15, 2024. To view the most recent and complete version of the guideline, go online to NCCN.org.

[7] Gordan JD , Kennedy EB, Abou-Alfa GK, et al Systemic therapy for advanced hepatocellular carcinoma: ASCO guideline update. J Clin Oncol. 2024;42:1830-1850. 10.1200/jco.23.02745 38502889

[8] Vogel A , Martinelli E, Vogel A, et al Updated treatment recommendations for hepatocellular carcinoma (HCC) from the ESMO clinical practice guidelines. Ann Oncol. 2021;32:801-805. 10.1016/j.annonc.2021.02.014 33716105

[9] Chen L-T , Martinelli E, Cheng A-L, et al Pan-Asian adapted ESMO clinical practice guidelines for the management of patients with intermediate and advanced/relapsed hepatocellular carcinoma: a TOS–ESMO initiative endorsed by CSCO, ISMPO, JSMO, KSMO, MOS and SSO. Ann Oncol. 2020;31:334-351. 10.1016/j.annonc.2019.12.001 32067677

[10] Celsa C , Cabibbo G, Pinato DJ, et al Balancing efficacy and tolerability of first-line systemic therapies for advanced hepatocellular carcinoma: a network meta-analysis. Liver Cancer. 2024;13:169-180. 10.1159/000531744 38751554 PMC11095611

[11] Eisenhauer EA , Therasse P, Bogaerts J, et al New response evaluation criteria in solid tumours: revised recist guideline (version 1.1). Eur J Cancer. 2009;45:228-247. 10.1016/j.ejca.2008.10.026 19097774

[12] Kulkarni AV , Tevethia H, Kumar K, et al Effectiveness and safety of atezolizumab-bevacizumab in patients with unresectable hepatocellular carcinoma: a systematic review and meta-analysis. EClinicalMedicine. 2023; 63:102179. https://www.ncbi.nlm.nih.gov/pmc/articles/PMC10480543/37680945 10.1016/j.eclinm.2023.102179 PMC10480543

[13] Shimose S , Saeki I, Tomonari T, et al Initial clinical experience with durvalumab plus tremelimumab in patients with unresectable hepatocellular carcinoma in real‑world practice. Oncol Lett. 2024;28:397. https://pubmed.ncbi.nlm.nih.gov/38979550/38979550 10.3892/ol.2024.14530 PMC11228928

[14] Lee P-C , Chao Y, Chen M-H, et al Predictors of response and survival in immune checkpoint inhibitor-treated unresectable hepatocellular carcinoma. Cancers (Basel). 2020;12:182. 10.3390/cancers12010182 31940757 PMC7017111

[15] Xie E , Yeo YH, Scheiner B, et al Immune checkpoint inhibitors for Child-Pugh class B advanced hepatocellular carcinoma. JAMA Oncol. 2023;9:1423-1431. https://jamanetwork.com/journals/jamaoncology/fullarticle/2808728 37615958 10.1001/jamaoncol.2023.3284 PMC10450588

[16] El Hajra I , Sanduzzi-Zamparelli M, Sapena V, et al Outcome of patients with HCC and liver dysfunction under immunotherapy: a systematic review and meta-analysis. Hepatology. 2023;77:1139-1149. https://pubmed.ncbi.nlm.nih.gov/36632997/36632997 10.1097/HEP.0000000000000030

[17] Tian B-W , Yan L-J, Ding Z-N, et al Evaluating liver function and the impact of immune checkpoint inhibitors in the prognosis of hepatocellular carcinoma patients: a systemic review and meta-analysis. Int Immunopharmacol. 2023;114:109519. https://pubmed.ncbi.nlm.nih.gov/36459922/36459922 10.1016/j.intimp.2022.109519

[18] Peter Robert G , Decaens T, Kudo M, et al Nivolumab (NIVO) plus ipilimumab (IPI) vs lenvatinib (LEN) or sorafenib (SOR) as first-line treatment for unresectable hepatocellular carcinoma (uHCC): first results from CheckMate 9DW. J Clin Oncol. 2024;42:LBA4008. 10.1200/jco.2024.42.17_suppl.lba4008

[19] Vogel A , Chan SL, Ren Z, et al Camrelizumab plus rivoceranib vs sorafenib as first-line therapy for unresectable hepatocellular carcinoma (uHCC): final overall survival analysis of the phase 3 CARES-310 study. J Clin Oncol. 2024;42:4110. 10.1200/jco.2024.42.16_suppl.4110

[20] Marell P , Kournoutas I, Gile J, et al Second line therapies in advanced hepatocellular carcinoma following first line atezolizumab and bevacizumab: multicenter single institution cohort experience. Oncologist. 2025;30:oyae342. 10.1093/oncolo/oyae342 39674576 PMC12396946

[21] Peng X , Gong C, Zhang W, Zhou A. Advanced development of biomarkers for immunotherapy in hepatocellular carcinoma. Front Oncol. 2022;12:1091088. 10.3389/fonc.2022.1091088 36727075 PMC9885011

[22] Pfister D , Núñez NG, Pinyol R, et al NASH limits anti-tumour surveillance in immunotherapy-treated HCC. Nature. 2021;592:450-456. 10.1038/s41586-021-03362-0 33762733 PMC8046670

[23] Kelley RK , Greten TF. Hepatocellular Carcinoma–Origins and Outcomes. Phimister EG, ed. N Engl J Med. 2021;385:280-282. 10.1056/nejmcibr2106594 34260842

[24] Lee C , Chan SL, Chon HJ. Could We predict the response of immune checkpoint inhibitor treatment in hepatocellular carcinoma? Cancers (Basel). 2022;14:3213-3213. 10.3390/cancers14133213 35804984 PMC9264773

[25] Kudo M. Lack of response to immunotherapy in non-alcoholic teatohepatitis related hepatocellular carcinoma. Hepatobiliary Surg Nutr. 2021;10:522-525. 10.21037/hbsn-21-203 34430534 PMC8350993

[26] Ding Z , Dong Z, Chen Z, et al Viral status and efficacy of immunotherapy in hepatocellular carcinoma: a systematic review with meta-analysis. Front Immunol. 2021;12:733530. 10.3389/fimmu.2021.733530 34659220 PMC8511422

[27] Shu DH , Ho WJ, Kagohara LT, et al Immunotherapy response induces divergent tertiary lymphoid structure morphologies in hepatocellular carcinoma. Nat Immunol. 2024;25:2110-2123. 10.1038/s41590-024-01992-w 39455893 PMC12042221

[28] Yang J , He J, Feng Y, Xiang M. Obesity contributes to hepatocellular carcinoma development via immunosuppressive microenvironment remodeling. Front Immunol. 2023;14:1166440. https://www.ncbi.nlm.nih.gov/pmc/articles/PMC10231659/37266440 10.3389/fimmu.2023.1166440 PMC10231659

[29] Eskridge W , Cryer DR, Schattenberg JM, et al Metabolic Dysfunction-associated steatotic liver disease and metabolic dysfunction-associated steatohepatitis: the patient and physician perspective. J Clin Med. 2023;12:6216. 10.3390/jcm12196216 37834859 PMC10573476

[30] Estes C , Anstee QM, Arias-Loste MT, et al Modeling NAFLD disease burden in China, France, Germany, Italy, Japan, Spain, United Kingdom, and United States for the period 2016–2030. J Hepatol. 2018;69:896-904. 10.1016/j.jhep.2018.05.036 29886156

[31] McQuade JL , Daniel CR, Hess KR, et al Association of body-mass index and outcomes in patients with metastatic melanoma treated with targeted therapy, immunotherapy, or chemotherapy: a retrospective, multicohort analysis. Lancet Oncol. 2018;19:310-322. https://pubmed.ncbi.nlm.nih.gov/29449192/29449192 10.1016/S1470-2045(18)30078-0 PMC5840029

[32] Wang Z , Aguilar EG, Luna JI, et al Paradoxical effects of obesity on T cell function during tumor progression and PD-1 checkpoint blockade. Nat Med. 2019;25:141-151. https://pubmed.ncbi.nlm.nih.gov/30420753/30420753 10.1038/s41591-018-0221-5 PMC6324991

[33] Li Y , Li C, Wu G, et al The obesity paradox in patients with colorectal cancer: a systematic review and meta-analysis. Nutr Rev. 2022;80:1755-1768. https://pubmed.ncbi.nlm.nih.gov/35182150/35182150 10.1093/nutrit/nuac005

[34] Antoun S , Lanoy E, Ammari S, et al Protective effect of obesity on survival in cancers treated with immunotherapy vanishes when controlling for type of cancer, weight loss and reduced skeletal muscle. Eur J Cancer. 2023;178:49-59. 10.1016/j.ejca.2022.10.013 36403367

[35] Uojima H , Chuma M, Hidaka H, et al Impact of body composition for patients with hepatocellular carcinoma who received atezolizumab plus bevacizumab therapy. Eur J Gastroenterol Hepatol. 2023;35:865-873. https://pubmed.ncbi.nlm.nih.gov/37395239/37395239 10.1097/MEG.0000000000002581

[36] Xu W , Yang Y, Yu Y, et al A multidimensional analysis of the impact of obesity on immune checkpoint inhibitor therapy efficacy. Cancer Cell Int. 2024;24:358. 10.1186/s12935-024-03532-w 39472922 PMC11523605

