# Supplementary material for: First-line atezolizumab/bevacizumab or durvalumab/tremelimumab in advanced hepatocellular carcinoma: a real world, multicenter retrospective study
Source: Oncologist. 2025 Sep 18;30(11):oyaf286. doi: 10.1093/oncolo/oyaf286 (PMC12604940; doi:10.1093/oncolo/oyaf286)
Supplement: oyaf286_Supplementary_Data [file oyaf286_supplementary_data.zip › Supplemental Table 12.docx]

# Supplemental Table 12, Multivariable adjusted disease control by obesity status

| **Variable** | **Odds Ratio** | **OR Lower CL** | **OR Upper CL** | **Pr > ChiSq** |
| --- | --- | --- | --- | --- |
| BMI, ≥30 kg/m2 vs <30 kg/m2 | 0.893 | 0.578 | 1.379 | 0.6092 |
| Age at Start of First Line | 1.008 | 0.988 | 1.030 | 0.4286 |
| Sex, Female vs Male | 0.667 | 0.412 | 1.079 | 0.0992 |
| Race, Non-White vs White | 0.885 | 0.512 | 1.529 | 0.6610 |
| Etiology, Viral vs Non-Viral | 0.667 | 0.426 | 1.045 | 0.0769 |
| Child-Pugh Class, B and C vs A | 0.468 | 0.291 | 0.754 | 0.0018 |
| Cirrhosis, Yes vs No | 1.671 | 0.999 | 2.794 | 0.0504 |
| ECOG |  |  |  | 0.6614* |
| ECOG, 1 vs 0 | 0.932 | 0.596 | 1.457 | 0.7566 |
| ECOG, 2 and 3 vs 0 | 0.710 | 0.338 | 1.489 | 0.3644 |
| Prior SIRT, Yes vs No | 2.305 | 1.109 | 4.791 | 0.0253 |

BMI: body mass index; ECOG: Eastern cooperative oncology group; SIRT: selective internal radiation therapy; *overall p-value for the multi-level categorical variable
