# Supplementary material for: First-line atezolizumab/bevacizumab or durvalumab/tremelimumab in advanced hepatocellular carcinoma: a real world, multicenter retrospective study
Source: Oncologist. 2025 Sep 18;30(11):oyaf286. doi: 10.1093/oncolo/oyaf286 (PMC12604940; doi:10.1093/oncolo/oyaf286)
Supplement: oyaf286_Supplementary_Data [file oyaf286_supplementary_data.zip › Supplemental Table 1.docx]

# Supplemental Table 1, Multivariable adjusted overall survival by first line agent

| **Variable** | **Hazard Ratio** | **HR Lower CL** | **HR Upper CL** | **Pr > ChiSq** |
| --- | --- | --- | --- | --- |
| Agent, Durva/Treme vs Atezo/Bev | 0.929 | 0.656 | 1.316 | 0.6790 |
| Age at Start of First Line | 0.998 | 0.984 | 1.013 | 0.8211 |
| Sex, Female vs Male | 1.383 | 1.011 | 1.891 | 0.0425 |
| Race, Non-White vs White | 0.755 | 0.516 | 1.105 | 0.1484 |
| Etiology, Viral vs Non-Viral | 1.134 | 0.853 | 1.508 | 0.3871 |
| Child-Pugh |  |  |  | <.0001* |
| Child-Pugh at First Line, B7 vs A | 2.002 | 1.365 | 2.937 | 0.0004 |
| Child-Pugh at First Line, B8 & B9 vs A | 2.182 | 1.373 | 3.468 | 0.0010 |
| Child-Pugh at First Line, C vs A | 6.096 | 2.863 | 12.978 | <.0001 |
| ALBI Grade |  |  |  | 0.0009* |
| ALBI Grade at First Line, A2 vs A1 | 1.989 | 1.383 | 2.861 | 0.0002 |
| ALBI Grade at First Line, A3 vs A1 | 2.235 | 1.195 | 4.180 | 0.0118 |
| Cirrhosis, Yes vs No | 0.830 | 0.570 | 1.210 | 0.3326 |
| ECOG |  |  |  | 0.0003* |
| ECOG, 1 vs 0 | 1.412 | 1.049 | 1.900 | 0.0227 |
| ECOG, 2 & 3 vs 0 | 2.589 | 1.624 | 4.127 | <.0001 |
| Prior SIRT, Yes vs No | 0.768 | 0.493 | 1.196 | 0.2422 |

Atezo/Bev: atezolizumab/bevacizumab; Durva/Treme: durvalumab/tremelimumab; ALBI: albumin-bilirubin; ECOG: Eastern cooperative oncology group; SIRT: selective internal radiation therapy; *overall p-value for the multi-level categorical variable
