# Supplementary material for: First-line atezolizumab/bevacizumab or durvalumab/tremelimumab in advanced hepatocellular carcinoma: a real world, multicenter retrospective study
Source: Oncologist. 2025 Sep 18;30(11):oyaf286. doi: 10.1093/oncolo/oyaf286 (PMC12604940; doi:10.1093/oncolo/oyaf286)
Supplement: oyaf286_Supplementary_Data [file oyaf286_supplementary_data.zip › Supplemental Table 2.docx]

# Supplemental Table 2, Multivariable adjusted time to treatment discontinuation by first line agent

| **Variable** | **Hazard Ratio** | **HR Lower CL** | **HR Upper CL** | **Pr > ChiSq** |
| --- | --- | --- | --- | --- |
| Agent, Durva/Treme vs Atezo/Bev | 1.067 | 0.814 | 1.398 | 0.6389 |
| Age at Start of First Line | 0.996 | 0.985 | 1.008 | 0.5646 |
| Sex, Female vs Male | 1.304 | 1.012 | 1.680 | 0.0401 |
| Race, Non-White vs White | 0.916 | 0.680 | 1.232 | 0.5611 |
| Etiology, Viral vs Non-Viral | 1.103 | 0.875 | 1.391 | 0.4057 |
| Child-Pugh |  |  |  | 0.0030* |
| Child-Pugh at First Line, B7 vs A | 1.583 | 1.151 | 2.175 | 0.0047 |
| Child-Pugh at First Line, B8 & B9 vs A | 1.611 | 1.083 | 2.397 | 0.0186 |
| Child-Pugh at First Line, C vs A | 2.790 | 1.364 | 5.708 | 0.0050 |
| ALBI Grade |  |  |  | 0.0101* |
| ALBI Grade at First Line, A2 vs A1 | 1.514 | 1.158 | 1.980 | 0.0024 |
| ALBI Grade at First Line, A3 vs A1 | 1.513 | 0.891 | 2.569 | 0.1250 |
| Cirrhosis, Yes vs No | 0.942 | 0.703 | 1.261 | 0.6867 |
| ECOG |  |  |  | 0.1428* |
| ECOG, 1 vs 0 | 0.946 | 0.750 | 1.193 | 0.6400 |
| ECOG, 2 & 3 vs 0 | 1.386 | 0.936 | 2.053 | 0.1035 |
| Prior SIRT, Yes vs No | 0.652 | 0.456 | 0.931 | 0.0188 |

Atezo/Bev: atezolizumab/bevacizumab; Durva/Treme: durvalumab/tremelimumab; ALBI: albumin-bilirubin; ECOG: Eastern cooperative oncology group; SIRT: selective internal radiation therapy; *overall p-value for the multi-level categorical variable
