# Supplementary material for: First-line atezolizumab/bevacizumab or durvalumab/tremelimumab in advanced hepatocellular carcinoma: a real world, multicenter retrospective study
Source: Oncologist. 2025 Sep 18;30(11):oyaf286. doi: 10.1093/oncolo/oyaf286 (PMC12604940; doi:10.1093/oncolo/oyaf286)
Supplement: oyaf286_Supplementary_Data [file oyaf286_supplementary_data.zip › Supplemental Table 3.docx]

# Supplemental Table 3, Multivariable adjusted objective response by first line agent

| **Variable** | **Odds Ratio** | **OR Lower CL** | **OR Upper CL** | **Pr > ChiSq** |
| --- | --- | --- | --- | --- |
| Agent, Durva/Treme vs Atezo/Bev | 0.721 | 0.410 | 1.267 | 0.2552 |
| Age at Start of First Line | 1.005 | 0.982 | 1.028 | 0.6861 |
| Sex, Female vs Male | 0.537 | 0.296 | 0.976 | 0.0412 |
| Race, Non-White vs White | 1.291 | 0.702 | 2.376 | 0.4110 |
| Etiology, Viral vs Non-Viral | 0.678 | 0.409 | 1.123 | 0.1310 |
| Child-Pugh Class, B and C vs A | 0.377 | 0.206 | 0.690 | 0.0016 |
| Cirrhosis, Yes vs No | 1.209 | 0.687 | 2.126 | 0.5101 |
| ECOG |  |  |  | 0.4155* |
| ECOG, 1 vs 0 | 0.903 | 0.554 | 1.472 | 0.6825 |
| ECOG, 2 and 3 vs 0 | 1.557 | 0.668 | 3.629 | 0.3052 |
| Prior SIRT, Yes vs No | 1.742 | 0.884 | 3.434 | 0.1088 |

Atezo/Bev: atezolizumab/bevacizumab; Durva/Treme: durvalumab/tremelimumab; ECOG: Eastern cooperative oncology group; SIRT: selective internal radiation therapy; *overall p-value for the multi-level categorical variable
