# Supplementary material for: First-line atezolizumab/bevacizumab or durvalumab/tremelimumab in advanced hepatocellular carcinoma: a real world, multicenter retrospective study
Source: Oncologist. 2025 Sep 18;30(11):oyaf286. doi: 10.1093/oncolo/oyaf286 (PMC12604940; doi:10.1093/oncolo/oyaf286)
Supplement: oyaf286_Supplementary_Data [file oyaf286_supplementary_data.zip › Supplemental Table 4.docx]

# Table 6, Multivariable adjusted disease control by first line agent

| **Variable** | **Odds Ratio** | **OR Lower CL** | **OR Upper CL** | **Pr > ChiSq** |
| --- | --- | --- | --- | --- |
| Agent, Durva/Treme vs Atezo/Bev | 0.741 | 0.456 | 1.204 | 0.2262 |
| Age at Start of First Line | 1.010 | 0.989 | 1.031 | 0.3605 |
| Sex, Female vs Male | 0.665 | 0.412 | 1.073 | 0.0950 |
| Race, Non-White vs White | 0.917 | 0.532 | 1.580 | 0.7544 |
| Etiology, Viral vs Non-Viral | 0.690 | 0.444 | 1.072 | 0.0990 |
| Child-Pugh Class, B and C vs A | 0.474 | 0.295 | 0.762 | 0.0021 |
| Cirrhosis, Yes vs No | 1.741 | 1.040 | 2.917 | 0.0350 |
| ECOG |  |  |  | 0.6889* |
| ECOG, 1 vs 0 | 0.932 | 0.599 | 1.452 | 0.7566 |
| ECOG, 2 and 3 vs 0 | 0.723 | 0.346 | 1.511 | 0.3884 |
| Prior SIRT, Yes vs No | 2.394 | 1.152 | 4.974 | 0.0193 |

Atezo/Bev: atezolizumab/bevacizumab; Durva/Treme: durvalumab/tremelimumab; ECOG: Eastern cooperative oncology group; SIRT: selective internal radiation therapy; *overall p-value for the multi-level categorical variable
