# Supplementary material for: First-line atezolizumab/bevacizumab or durvalumab/tremelimumab in advanced hepatocellular carcinoma: a real world, multicenter retrospective study
Source: Oncologist. 2025 Sep 18;30(11):oyaf286. doi: 10.1093/oncolo/oyaf286 (PMC12604940; doi:10.1093/oncolo/oyaf286)
Supplement: oyaf286_Supplementary_Data [file oyaf286_supplementary_data.zip › Supplemental Table 5.docx]

# Supplemental Table 5, Multivariable adjusted overall survival by first line agent excluding patients with durvalumab monotherapy

| **Variable** | **Hazard Ratio** | **HR Lower CL** | **HR Upper CL** | **Pr > ChiSq** |
| --- | --- | --- | --- | --- |
| Agent, Durva/Treme vs Atezo/Bev | 1.047 | 0.714 | 1.535 | 0.8152 |
| Age at Start of First Line | 0.997 | 0.981 | 1.012 | 0.6593 |
| Sex, Female vs Male | 1.344 | 0.978 | 1.848 | 0.0687 |
| Race, Non-White vs White | 0.731 | 0.495 | 1.079 | 0.1144 |
| Etiology, Viral vs Non-Viral | 1.184 | 0.884 | 1.587 | 0.2573 |
| Child-Pugh |  |  |  | <.0001* |
| Child-Pugh at First Line, B7 vs A | 2.180 | 1.472 | 3.230 | 0.0001 |
| Child-Pugh at First Line, B8 & B9 vs A | 2.326 | 1.461 | 3.702 | 0.0004 |
| Child-Pugh at First Line, C vs A | 7.023 | 3.174 | 15.540 | <.0001 |
| ALBI Grade |  |  |  | 0.0034* |
| ALBI Grade at First Line, A2 vs A1 | 1.890 | 1.296 | 2.757 | 0.0009 |
| ALBI Grade at First Line, A3 vs A1 | 2.228 | 1.182 | 4.202 | 0.0133 |
| Cirrhosis, Yes vs No | 0.814 | 0.556 | 1.190 | 0.2876 |
| ECOG |  |  |  | 0.0004* |
| ECOG, 1 vs 0 | 1.418 | 1.040 | 1.933 | 0.0273 |
| ECOG, 2 & 3 vs 0 | 2.611 | 1.622 | 4.202 | <.0001 |
| Prior SIRT, Yes vs No | 0.758 | 0.478 | 1.201 | 0.2377 |

Atezo/Bev: atezolizumab/bevacizumab; Durva/Treme: durvalumab/tremelimumab; ALBI: albumin-bilirubin; ECOG: Eastern cooperative oncology group; SIRT: selective internal radiation therapy; *overall p-value for the multi-level categorical variable
