# Supplementary material for: First-line atezolizumab/bevacizumab or durvalumab/tremelimumab in advanced hepatocellular carcinoma: a real world, multicenter retrospective study
Source: Oncologist. 2025 Sep 18;30(11):oyaf286. doi: 10.1093/oncolo/oyaf286 (PMC12604940; doi:10.1093/oncolo/oyaf286)
Supplement: oyaf286_Supplementary_Data [file oyaf286_supplementary_data.zip › Supplemental Table 6.docx]

# Supplemental Table 6, Multivariable adjusted time to treatment discontinuation by first line agent excluding patients with durvalumab monotherapy

| **Variable** | **Hazard Ratio** | **HR Lower CL** | **HR Upper CL** | **Pr > ChiSq** |
| --- | --- | --- | --- | --- |
| Agent, Durva +/- Treme vs Atezo/Bev | 1.118 | 0.833 | 1.500 | 0.4593 |
| Age at Start of First Line | 0.995 | 0.983 | 1.007 | 0.4201 |
| Sex, Female vs Male | 1.284 | 0.992 | 1.660 | 0.0574 |
| Race, Non-White vs White | 0.907 | 0.668 | 1.231 | 0.5300 |
| Etiology, Viral vs Non-Viral | 1.123 | 0.885 | 1.424 | 0.3392 |
| Child-Pugh |  |  |  | 0.0005* |
| Child-Pugh at First Line, B7 vs A | 1.718 | 1.237 | 2.387 | 0.0012 |
| Child-Pugh at First Line, B8 & B9 vs A | 1.676 | 1.123 | 2.501 | 0.0115 |
| Child-Pugh at First Line, C vs A | 3.371 | 1.596 | 7.121 | 0.0014 |
| ALBI Grade |  |  |  | 0.0510* |
| ALBI Grade at First Line, A2 vs A1 | 1.412 | 1.068 | 1.867 | 0.0154 |
| ALBI Grade at First Line, A3 vs A1 | 1.490 | 0.869 | 2.553 | 0.1469 |
| Cirrhosis, Yes vs No | 0.937 | 0.698 | 1.258 | 0.6633 |
| ECOG |  |  |  | 0.1071* |
| ECOG, 1 vs 0 | 0.962 | 0.757 | 1.222 | 0.7489 |
| ECOG, 2 & 3 vs 0 | 1.453 | 0.975 | 2.166 | 0.0663 |
| Prior SIRT, Yes vs No | 0.673 | 0.467 | 0.970 | 0.0339 |

Atezo/Bev: atezolizumab/bevacizumab; Durva/Treme: durvalumab/tremelimumab; ALBI: albumin-bilirubin; ECOG: Eastern cooperative oncology group; SIRT: selective internal radiation therapy; *overall p-value for the multi-level categorical variable
