# Supplementary material for: First-line atezolizumab/bevacizumab or durvalumab/tremelimumab in advanced hepatocellular carcinoma: a real world, multicenter retrospective study
Source: Oncologist. 2025 Sep 18;30(11):oyaf286. doi: 10.1093/oncolo/oyaf286 (PMC12604940; doi:10.1093/oncolo/oyaf286)
Supplement: oyaf286_Supplementary_Data [file oyaf286_supplementary_data.zip › Supplemental Table 7.docx]

# Supplemental Table 7, Multivariable adjusted objective response by first line agent excluding patients with durvalumab monotherapy

| **Variable** | **Odds Ratio** | **OR Lower CL** | **OR Upper CL** | **Pr > ChiSq** |
| --- | --- | --- | --- | --- |
| Agent, Durva +/- Treme vs Atezo/Bev | 0.782 | 0.429 | 1.425 | 0.4217 |
| Age at Start of First Line | 1.007 | 0.984 | 1.031 | 0.5540 |
| Sex, Female vs Male | 0.507 | 0.275 | 0.935 | 0.0296 |
| Race, Non-White vs White | 1.182 | 0.634 | 2.202 | 0.5985 |
| Etiology, Viral vs Non-Viral | 0.672 | 0.402 | 1.123 | 0.1290 |
| Child-Pugh Class, B and C vs A | 0.372 | 0.201 | 0.688 | 0.0016 |
| Cirrhosis, Yes vs No | 1.223 | 0.694 | 2.156 | 0.4858 |
| ECOG |  |  |  | 0.5370* |
| ECOG, 1 vs 0 | 0.848 | 0.516 | 1.392 | 0.5140 |
| ECOG, 2 and 3 vs 0 | 1.311 | 0.550 | 3.125 | 0.5411 |
| Prior SIRT, Yes vs No | 1.669 | 0.830 | 3.355 | 0.1504 |

Atezo/Bev: atezolizumab/bevacizumab; Durva/Treme: durvalumab/tremelimumab; ECOG: Eastern cooperative oncology group; SIRT: selective internal radiation therapy; *overall p-value for the multi-level categorical variable
