# Supplementary material for: First-line atezolizumab/bevacizumab or durvalumab/tremelimumab in advanced hepatocellular carcinoma: a real world, multicenter retrospective study
Source: Oncologist. 2025 Sep 18;30(11):oyaf286. doi: 10.1093/oncolo/oyaf286 (PMC12604940; doi:10.1093/oncolo/oyaf286)
Supplement: oyaf286_Supplementary_Data [file oyaf286_supplementary_data.zip › Supplemental Table 8.docx]

# Supplemental Table 8, Multivariable adjusted disease control by first line agent excluding patients with durvalumab monotherapy

| **Variable** | **Odds Ratio** | **OR Lower CL** | **OR Upper CL** | **Pr > ChiSq** |
| --- | --- | --- | --- | --- |
| Agent, Durva +/- Treme vs Atezo/Bev | 0.654 | 0.389 | 1.099 | 0.1088 |
| Age at Start of First Line | 1.014 | 0.993 | 1.035 | 0.2055 |
| Sex, Female vs Male | 0.658 | 0.404 | 1.070 | 0.0918 |
| Race, Non-White vs White | 0.969 | 0.556 | 1.687 | 0.9108 |
| Etiology, Viral vs Non-Viral | 0.647 | 0.412 | 1.017 | 0.0589 |
| Child-Pugh Class, B and C vs A | 0.416 | 0.255 | 0.678 | 0.0004 |
| Cirrhosis, Yes vs No | 1.812 | 1.075 | 3.054 | 0.0257 |
| ECOG |  |  |  | 0.7359* |
| ECOG, 1 vs 0 | 0.959 | 0.608 | 1.511 | 0.8567 |
| ECOG, 2 and 3 vs 0 | 0.744 | 0.351 | 1.578 | 0.4410 |
| Prior SIRT, Yes vs No | 2.238 | 1.065 | 4.704 | 0.0335 |

Atezo/Bev: atezolizumab/bevacizumab; Durva/Treme: durvalumab/tremelimumab; ECOG: Eastern cooperative oncology group; SIRT: selective internal radiation therapy; *overall p-value for the multi-level categorical variable
