# Supplementary material for: First-line atezolizumab/bevacizumab or durvalumab/tremelimumab in advanced hepatocellular carcinoma: a real world, multicenter retrospective study
Source: Oncologist. 2025 Sep 18;30(11):oyaf286. doi: 10.1093/oncolo/oyaf286 (PMC12604940; doi:10.1093/oncolo/oyaf286)
Supplement: oyaf286_Supplementary_Data [file oyaf286_supplementary_data.zip › Supplemental Table 9.docx]

# Supplemental Table 9, Multivariable adjusted overall survival by obesity status

| **Variable** | **Hazard Ratio** | **HR Lower CL** | **HR Upper CL** | **Pr > ChiSq** |
| --- | --- | --- | --- | --- |
| BMI, ≥30 kg/m2 vs <30 kg/m2 | 0.815 | 0.616 | 1.078 | 0.1520 |
| Age at Start of First Line | 0.998 | 0.984 | 1.013 | 0.8140 |
| Sex, Female vs Male | 1.360 | 0.993 | 1.862 | 0.0550 |
| Race, Non-White vs White | 0.725 | 0.499 | 1.054 | 0.0918 |
| Etiology, Viral vs Non-Viral | 1.096 | 0.822 | 1.460 | 0.5336 |
| Child-Pugh |  |  |  | <.0001* |
| Child-Pugh at First Line, B7 vs A | 1.987 | 1.354 | 2.917 | 0.0004 |
| Child-Pugh at First Line, B8 & B9 vs A | 2.224 | 1.403 | 3.526 | 0.0007 |
| Child-Pugh at First Line, C vs A | 6.148 | 2.881 | 13.117 | <.0001 |
| ALBI |  |  |  | 0.0010* |
| ALBI Grade at First Line, A2 vs A1 | 1.981 | 1.376 | 2.851 | 0.0002 |
| ALBI Grade at First Line, A3 vs A1 | 2.217 | 1.191 | 4.126 | 0.0120 |
| Cirrhosis, Yes vs No | 0.845 | 0.580 | 1.230 | 0.3794 |
| ECOG |  |  |  | 0.0001* |
| ECOG, 1 vs 0 | 1.468 | 1.086 | 1.985 | 0.0125 |
| ECOG, 2 & 3 vs 0 | 2.697 | 1.689 | 4.307 | <.0001 |
| Prior SIRT, Yes vs No | 0.781 | 0.501 | 1.218 | 0.2756 |

BMI: body mass index; ALBI: albumin-bilirubin; ECOG: Eastern cooperative oncology group; SIRT: selective internal radiation therapy; *overall p-value for the multi-level categorical variable
