# Supplementary material for: First-line atezolizumab/bevacizumab or durvalumab/tremelimumab in advanced hepatocellular carcinoma: a real world, multicenter retrospective study
Source: Oncologist. 2025 Sep 18;30(11):oyaf286. doi: 10.1093/oncolo/oyaf286 (PMC12604940; doi:10.1093/oncolo/oyaf286)
Supplement: oyaf286_Supplementary_Data [file oyaf286_supplementary_data.zip › Supplemental Table 10.docx]

# Supplemental Table 10, Multivariable adjusted time to treatment discontinuation by obesity status

| **Variable** | **Hazard Ratio** | **HR Lower CL** | **HR Upper CL** | **Pr > ChiSq** |
| --- | --- | --- | --- | --- |
| BMI, ≥30 kg/m2 vs <30 kg/m2 | 0.902 | 0.721 | 1.128 | 0.3651 |
| Age at Start of First Line | 0.996 | 0.985 | 1.008 | 0.5551 |
| Sex, Female vs Male | 1.289 | 1.000 | 1.663 | 0.0504 |
| Race, Non-White vs White | 0.923 | 0.691 | 1.232 | 0.5862 |
| Etiology, Viral vs Non-Viral | 1.094 | 0.865 | 1.382 | 0.4538 |
| Child-Pugh |  |  |  | 0.0022* |
| Child-Pugh at First Line, B7 vs A | 1.600 | 1.162 | 2.202 | 0.0039 |
| Child-Pugh at First Line, B8 & B9 vs A | 1.625 | 1.093 | 2.416 | 0.0164 |
| Child-Pugh at First Line, C vs A | 2.906 | 1.422 | 5.940 | 0.0034 |
| ALBI Grade |  |  |  | 0.0132* |
| ALBI Grade at First Line, A2 vs A1 | 1.498 | 1.144 | 1.962 | 0.0033 |
| ALBI Grade at First Line, A3 vs A1 | 1.514 | 0.894 | 2.565 | 0.1229 |
| Cirrhosis, Yes vs No | 0.950 | 0.710 | 1.271 | 0.7314 |
| ECOG |  |  |  | 0.1239* |
| ECOG, 1 vs 0 | 0.965 | 0.763 | 1.220 | 0.7650 |
| ECOG, 2 & 3 vs 0 | 1.430 | 0.964 | 2.123 | 0.0757 |
| Prior SIRT, Yes vs No | 0.664 | 0.465 | 0.948 | 0.0244 |

BMI: body mass index; ALBI: albumin-bilirubin; ECOG: Eastern cooperative oncology group; SIRT: selective internal radiation therapy; *overall p-value for the multi-level categorical variable
