# Supplementary material for: First-line atezolizumab/bevacizumab or durvalumab/tremelimumab in advanced hepatocellular carcinoma: a real world, multicenter retrospective study
Source: Oncologist. 2025 Sep 18;30(11):oyaf286. doi: 10.1093/oncolo/oyaf286 (PMC12604940; doi:10.1093/oncolo/oyaf286)
Supplement: oyaf286_Supplementary_Data [file oyaf286_supplementary_data.zip › Supplemental Table 11.docx]

# Supplemental Table 11, Multivariable adjusted objective response by obesity status

| **Variable** | **Odds Ratio** | **OR Lower CL** | **OR Upper CL** | **Pr > ChiSq** |
| --- | --- | --- | --- | --- |
| BMI, ≥30 kg/m2 vs <30 kg/m2 | 0.719 | 0.441 | 1.173 | 0.1865 |
| Age at Start of First Line | 1.004 | 0.982 | 1.028 | 0.7058 |
| Sex, Female vs Male | 0.537 | 0.295 | 0.975 | 0.0412 |
| Race, Non-White vs White | 1.198 | 0.650 | 2.210 | 0.5622 |
| Etiology, Viral vs Non-Viral | 0.647 | 0.387 | 1.081 | 0.0962 |
| Child-Pugh Class, B and C vs A | 0.378 | 0.206 | 0.691 | 0.0016 |
| Cirrhosis, Yes vs No | 1.168 | 0.667 | 2.047 | 0.5861 |
| ECOG |  |  |  | 0.4369* |
| ECOG, 1 vs 0 | 0.942 | 0.575 | 1.544 | 0.8136 |
| ECOG, 2 and 3 vs 0 | 1.602 | 0.685 | 3.747 | 0.2773 |
| Prior SIRT, Yes vs No | 1.727 | 0.874 | 3.413 | 0.1157 |

BMI: body mass index; ECOG: Eastern cooperative oncology group; SIRT: selective internal radiation therapy; *overall p-value for the multi-level categorical variable
