# Supplementary figures and images for: First-line atezolizumab/bevacizumab or durvalumab/tremelimumab in advanced hepatocellular carcinoma: a real world, multicenter retrospective study
Source: Oncologist. 2025 Sep 18;30(11):oyaf286. doi: 10.1093/oncolo/oyaf286 (PMC12604940; doi:10.1093/oncolo/oyaf286)

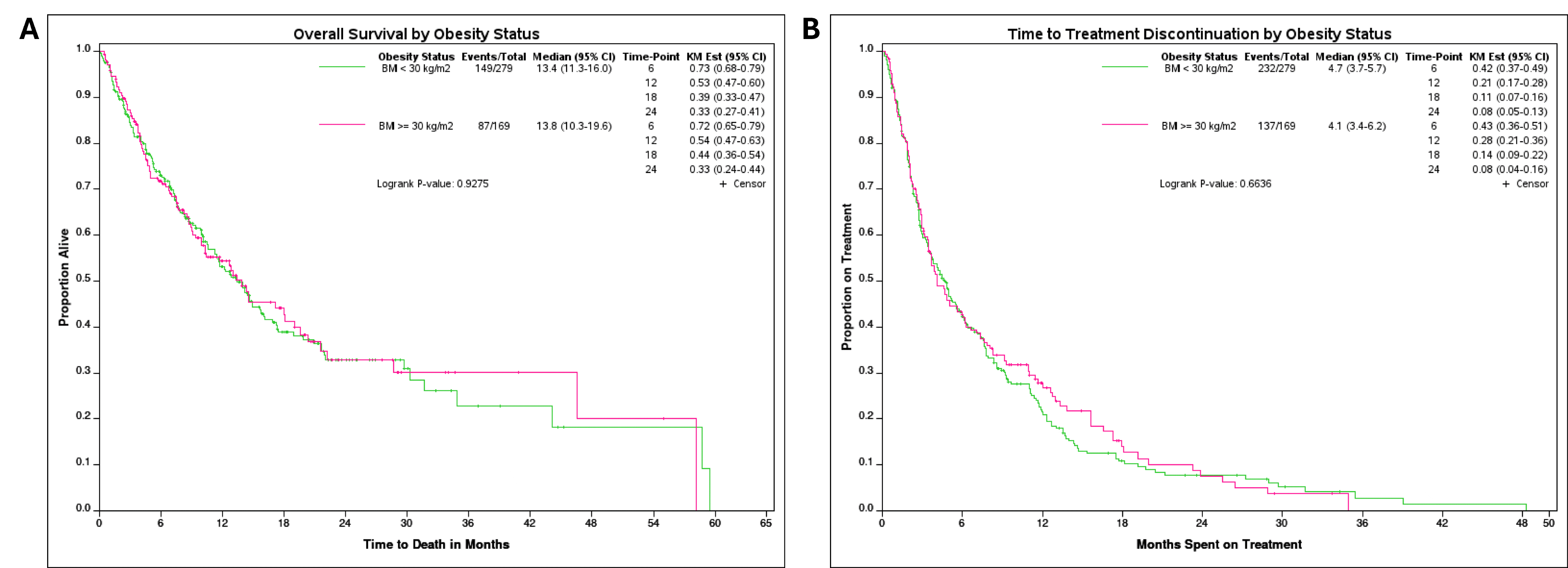

Supplement: oyaf286_Supplementary_Data [file oyaf286_supplementary_data.zip › Supplemental Figure 1a_1b.tif]
